# Improving bioinformatics software quality through teamwork

**DOI:** 10.1093/bioinformatics/btae632

**Published:** 2024-10-22

**Authors:** Katalin Ferenc, Ieva Rauluseviciute, Ladislav Hovan, Vipin Kumar, Marieke L Kuijjer, Anthony Mathelier

**Affiliations:** Centre for Molecular Medicine Norway (NCMM), Nordic EMBL Partnership, University of Oslo, Oslo 0318, Norway; Centre for Molecular Medicine Norway (NCMM), Nordic EMBL Partnership, University of Oslo, Oslo 0318, Norway; Centre for Molecular Medicine Norway (NCMM), Nordic EMBL Partnership, University of Oslo, Oslo 0318, Norway; Centre for Molecular Medicine Norway (NCMM), Nordic EMBL Partnership, University of Oslo, Oslo 0318, Norway; Centre for Molecular Medicine Norway (NCMM), Nordic EMBL Partnership, University of Oslo, Oslo 0318, Norway; Centre for Molecular Medicine Norway (NCMM), Nordic EMBL Partnership, University of Oslo, Oslo 0318, Norway; Department of Medical Genetics, Institute of Clinical Medicine, Oslo University Hospital and University of Oslo, Oslo 0450, Norway; Department of Pharmacy, University of Oslo, Oslo 0371, Norway

## Abstract

**Summary:**

Since high-throughput techniques became a staple in biological science laboratories, computational algorithms, and scientific software have boomed. However, the development of bioinformatics software usually lacks software development quality standards. The resulting software code is hard to test, reuse, and maintain. We believe that the root of inefficiency in implementing the best software development practices in academic settings is the individualistic approach, which has traditionally been the norm for recognizing scientific achievements and, by extension, for developing specialized software. Software development is a collective effort in most software-heavy endeavors. Indeed, the literature suggests teamwork directly impacts code quality through knowledge sharing, collective software development, and established coding standards. In our computational biology research groups, we sustainably involve all group members in learning, sharing, and discussing software development while maintaining the personal ownership of research projects and related software products. We found that group members involved in this endeavor improved their coding skills, became more efficient bioinformaticians, and obtained detailed knowledge about their peers’ work, triggering new collaborative projects. We strongly advocate for improving software development culture within bioinformatics through collective effort in computational biology groups or institutes with three or more bioinformaticians.

**Availability and implementation:**

Additional information and guidance on how to get started is available at https://ferenckata.github.io/ImprovingSoftwareTogether.github.io/.

## 1 Introduction

Bioinformatics and computational biology are integral to modern biological research (Markowetz 2017). However, bioinformatics software often lacks the quality standards found in other software-heavy fields, leading to reproducibility, maintenance, and efficiency issues. One implication of using outdated or poor software engineering practices is that incorrect software may result in invalid scientific findings (Miller 2006, Goble 2014, Noor 2022). Furthermore, such practice results in the accumulation of technical debt, which translates to increased future time investments in code refactoring and rework (Cunningham 1992, Allman 2012).

Adopting best practices from software engineering is essential to mitigate these issues (Noor 2022). Unfortunately, many bioinformaticians lack formal training in software development, which hinders the implementation of these practices (Hannay *et al.* 2009, Taschuk and Wilson 2017, Noor 2022). Furthermore, the current academic framework typically restricts research projects to individual trainees, limiting the usability of frameworks, tools, and processes that would support quality software development.

We propose that organizing bioinformaticians into collaborative teams at the scale of individual research groups or institutions can significantly enhance software quality. By setting the environment to leverage knowledge sharing, teamwork in reviewing code, and collective responsibility, bioinformaticians can achieve better code quality, reproducibility, and more sustainable software maintenance (Hannay *et al.* 2009, Russell *et al.* 2018). At our institute, we implemented this approach through regular (i) software quality seminars, (ii) code reviews, and (iii) resource sharing. This collectively improved our coding standards and efficiency. Thus, we believe that implementing structured collaborative efforts is essential for high-quality bioinformatics software development.

This manuscript details these critical efforts. We strongly advocate for creating a collaborative environment, ideally across research groups, to implement essential software development practices with shared knowledge. We emphasize that the underlying software development practices are beneficial and necessary for advancing computational biology. Our experience demonstrates that by working together bioinformaticians can overcome the inherent challenges of scientific software development, thus contributing more effectively to science.

## 2 Challenges with adopting good practices

Scientific software development practices have been critiqued for over 15 years. A 2007 landmark paper by Diane F. Kelly stated that scientific computations are performed using error-prone development practices and reaching sub-optimal solutions and poor software quality (Kelly 2007). Since then, software engineering researchers have surveyed and discussed the limitations and caveats of scientific software development practices and products (Segal and Morris 2008, Hannay *et al.* 2009, Umarji *et al.* 2009, Nguyen-Hoan *et al.* 2010, Goble 2014, Arvanitou *et al.* 2021, Ferenc *et al.* 2022). Despite this effort, scientific software development practices changed little, resulting in more recent papers repeating the same findings and suggestions (Noor 2022).

The persistence of this gap can be traced to the establishment of software engineering as an autonomous discipline (Kelly 2007). In doing so, the best practices, extensively field-tested by the software industry, were formulated in generic terms whose concrete relation to bioinformatics is left at the discretion of the particular practitioner (Segal and Morris 2008). Indeed, the field of bioinformatics has unique properties and culture that prevent the direct adoption of software industry practices. We do not believe that all the software engineering guidelines employed in the industry are necessarily relevant to producing scientific software in academia. We believe the main cultural differences are the variability in research project outcomes (e.g. papers, tools, protocols) and that the academic system encourages crediting individual researchers to aid their career progression.

In all software-heavy industries, the team is considered a basic unit in software development. Producing code in the context of a team implies enabling multiple developers to contribute to the same project effectively. This requires the effective adoption of dedicated software development methodologies that support the collective ownership and governance of the code base (Moe *et al.* 2010). In doing so, team coding serves as a deliberate context in which the adoption of many of the software engineering recommendations and best practices becomes necessary.

We argue that effectively adopting these practices requires our community to discuss them and adapt them to our purposes. Beyond practices, the actual tools aiding software development can rarely be used to their full potential by a single individual. One illustrative example of this is the usage of version control systems such as GitHub (http://github.com/), Bitbucket (https://bitbucket.org/), or GitLab (https://about.gitlab.com/), which has been recommended and discussed in previous papers (Nguyen-Hoan *et al.* 2010, Sandve *et al.* 2013, Blischak *et al.* 2016, Russell *et al.* 2018). Although it is established that the bioinformatics community benefits from adopting good practices and standard tools, individual bioinformaticians may not perceive significant added value because they bear the full burden of adoption. We believe that creating a learning and reviewing community would decrease this burden and increase the benefit at the individual’s level, thus contributing to the broader bioinformatics society.

Indeed, the particular context of academia should not prevent interactions between developers of distinct projects, as these interactions, analogously to team-based coding, will promote compliance with guidelines and increase software quality. Our proposition of building a learning community centered on coding is our attempt to bridge the gap between the merits of coding in teams and the reality of individually credited projects in the academic setting. While teamwork is crucial for improving software quality through knowledge sharing and collective efforts, it is important to ensure that individual researchers maintain personal ownership of their projects.

**Figure 1.**
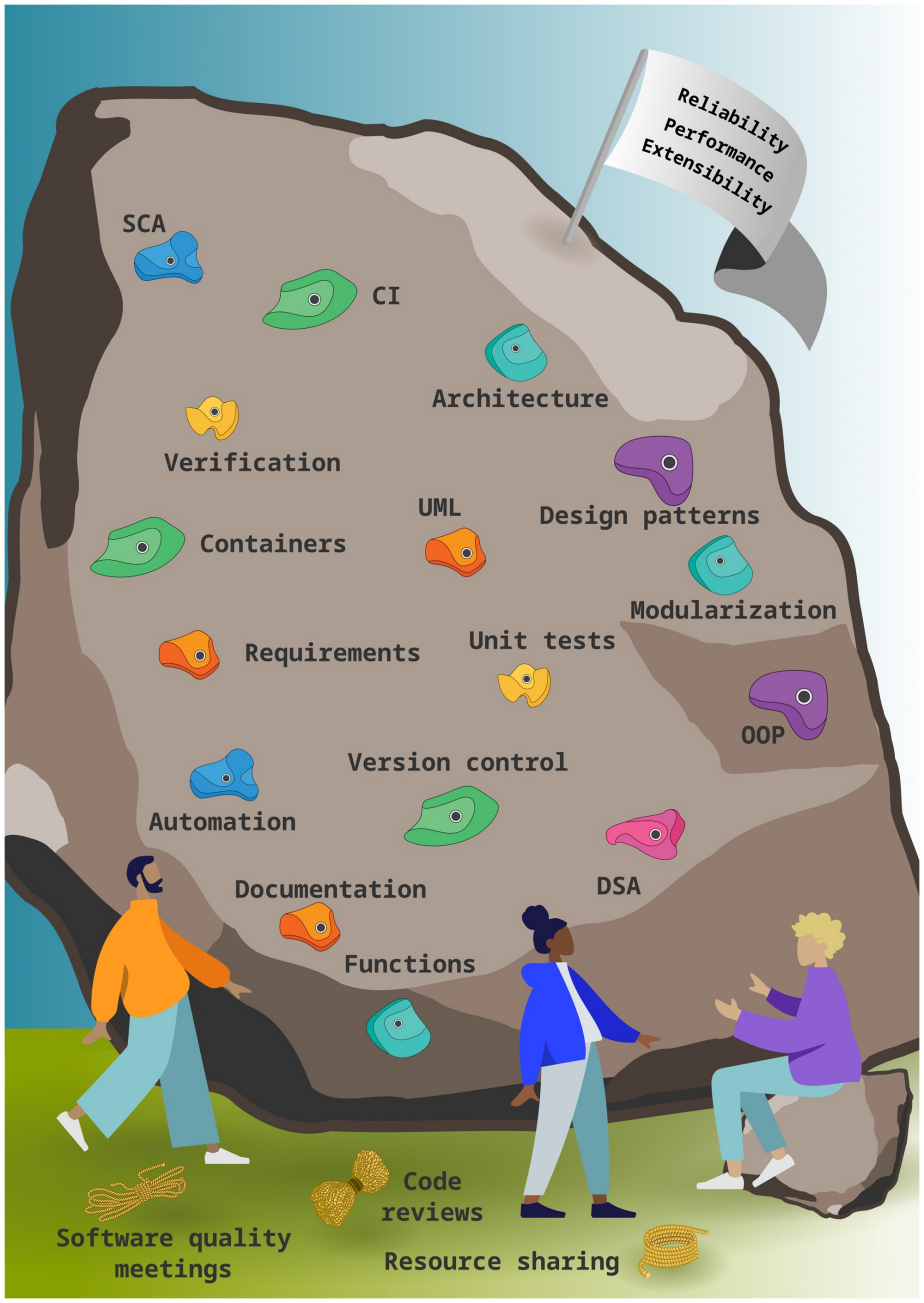
An illustration comparing the improvement process in software writing to rock climbing. *DSA*: data structures and algorithms, *OOP*: object-oriented programming, *UML*: unified modelling language, *CI*: continuous integration, *SCA*: static code analysis.

We draw a visual metaphor where improving software quality is similar to a rock climbing exercise (Fig. 1). One striking feature of sport climbing is the act of “reading the route” together, meaning that participants discuss the most optimal way to reach their goal before any one of them even starts climbing. Indeed, we believe selecting topics (from the literature or professional experience), deciding on the entry point, and deciding on how to approach them is an exercise that helps bridge the gap described above. The necessary process of composing your path involves assessing the reachability of holds. This, we argue, corresponds to determining the interdependencies between different software engineering concepts. The climbing itself is a team effort too, mediated by ropes between the participants. Such mediation would involve the meetings and processes we have introduced to foster team effort. Given the dynamic nature of academic teams and software development, we recommend having regular discussions so that the insights of new members and shifting priorities can be accommodated. We embrace revisiting topics, as it is a great way to deepen their understanding and ease of adoption.

In the following section, we share our experience developing such a learning community to enable the adoption of good software engineering practices in a bioinformatics academic setting.

## 3 Improving development processes as a team

Over the course of three years, we have grown from a team of 5 people from 2 research groups to a core of about 10 people from up to 5 research groups who regularly attend the meetings on average. In addition, we have about 50 people on the mailing list, some of whom attend occasionally for specific topics or when they have something to discuss. We have adopted improved software development practices by implementing three pillars: (i) software quality seminars, (ii) code review sessions, and (iii) resource sharing. We aim to hold the software quality seminars and code review sessions every week, but we do not specifically recommend this, as groups can adjust this schedule to their timelines and needs. We describe hereafter our experience and recommendations for implementing these pillars.

### 3.1 Software quality seminars

The software quality seminars are meant for transferring knowledge among participants to substitute or complement a more formal computer science education (Noor 2022). Each seminar is structured to build a shared vocabulary among members to facilitate discussions on implementation details and code structures. These seminars include presentations and demonstrations covering basic concepts. Beyond this, this platform introduces new techniques and showcases tools not limited to specific projects. These seminars help in three ways: broadening our collective knowledge, providing an opportunity to examine theoretical concepts in practice, and building a community by encouraging all members to present their topics of interest. While preparing the lectures can represent a significant time investment, this effort is rewarded by the substantial knowledge gained from attending each other’s presentations. Finally, the investment pays off in the long run since acquiring knowledge leads to greater efficiency and expertise in future projects. Overall, software quality seminars expose the members to new intuitions, technologies, and theoretical details.

### 3.2 Code review sessions

Code review is a standard process in the industry, but in the bioinformatics academic setting, it seems just to get its footing (Hagan *et al.* 2020). This might be due to the limited number of collaborative software projects in academia, which would necessitate the creative implementation of code reviews. The benefits of code reviews have been reviewed extensively (Hunt and Thomas 1999, Crusoe and Brown 2016, Hagan *et al.* 2020, Posner 2022). Some benefits, such as implementing consistent coding standards and detecting bugs/errors, are obvious. In contrast, others represent less expected outcomes, such as diverse learning, fostering a positive environment, and enhancing efficiency. Before a scheduled code review, developers are more likely to write their code in a way that others will understand, which improves code readability and structuring. Even within a friendly environment, this expectation is mostly self-inflicted. During a code review session, the developer must clearly explain some aspects of their code (e.g. structure, algorithm implementation, or performance-related decisions). We recommend that the developers decide on the aspect of the code they want to focus on during these sessions. While code review sessions generally focus on implementation details, they can trigger discussions on any aspect of the code, including architectural considerations, documentation, and user interface design.

Other participants in these sessions may not be deeply familiar with the project for which the code is developed. However, they can bring their complementary knowledge and viewpoints. The feedback obtained can help fix existing or avoid potential future issues, improve code implementation, and produce cleaner and more concise code. Our experience indicates a broader adoption of the theoretical aspects and good software engineering practices discussed during the software quality seminars. We found that the implicit soft peer pressure that came with these code review sessions successfully addressed most of our goals: standardization of practices, improved code quality, and enhanced software usability.

As a beneficial side effect, we observed an enhanced understanding of each other’s projects that naturally resulted from scrutinizing the code. It helped us understand the underlying scientific questions better and led to more insightful comments during subsequent group meetings. In addition, hands-on analysis of code revealed the repetitiveness of certain coding elements across projects. To address this redundancy, we recommend implementing a system to share resources.

### 3.3 Resource sharing

Resource sharing fundamentally involves ensuring that valuable resources are readily accessible to all participants. We efficiently implemented the sharing of two types of resources: External open-access resources (forums, repositories, packages, and libraries) and internal resources (recordings of the software quality seminar lectures, as well as tools developed within the groups). Sharing internal resources is crucial, fostering team contributions that can enhance individual project development. For instance, consider a shared repository containing various computational tools developed by multiple group members. These tools are universal and aligned with the group’s research questions. Furthermore, the group collectively develops and reviews the underlying code base, enhancing its utility and quality.

These three pillars engage members of our learning community to strengthen its sustainability and vitality. While these initiatives foster collaboration and shared responsibility, each researcher retains full ownership of their specific project and software, ensuring that personal achievements are recognized alongside collaborative efforts.

## 4 Conclusions

To summarize, we view the implementation of software quality seminars, code reviews, and shared resources in the academic setting as critical tools for improving software development and better trusting the resulting scientific discoveries. Nevertheless, we recognize that scientists can choose to implement all or any of them as independent activities. We observed that even a single activity benefits the members’ coding experience and the resulting code quality. As good practices become routine, the required time investment will decrease, and the benefits will become more apparent. Finally, the shared knowledge base and standards help new group members adopt good coding practices more quickly. To elaborate further, we created a resource page where we collected suggestions from the literature and discussed three concepts: the adoption of testing, modularization, and dependency management practices (https://ferenckata.github.io/ImprovingSoftwareTogether.github.io/).

As bioinformatics becomes increasingly software-heavy, we believe a good direction is to collectively lower the barrier to adapting to new technologies. Working in a team and following standards is necessary for large software projects that support many researchers and contribute to novel findings. We argue that following good software quality practices and mimicking team structure benefits small projects too. Therefore, we motivate all group leaders with a computational component in their research programs, whether small or large, to build an environment for their trainees to communicate and discuss software quality aspects. Likewise, we motivate all trainees of computational research teams to actively create an environment where they can discuss the quality of their scientific software solutions and communicate the need to their group leaders, too.

## 5 Future perspectives

We envision a future where scientific software for core applications is appreciated, reliable, and actively maintained. A strong backbone of software solutions that supports quick and efficient prototyping and the maturation of working solutions would benefit all scientists. Unfortunately, we recognize that the lack of funding for software maintenance prevents achieving a level of software quality that would inspire confidence in the results (Goble 2014). Funding agencies, often through peer reviewers, generally emphasize “novelty” by enforcing new software development. This focus can make it difficult to justify dedicating time to the maintenance of software or computational resources that offer limited immediate scientific output, especially when considering journal publications as the only token of success. As Alexander Szalay points out, a key difficulty of building a solid code base for scientific development is that “the funding stops when researchers develop the software prototype” (Matthews 2022).

Researchers want to build on each other’s findings and use published novel software as tools. Still, they often need to spend significant time adopting or maintaining that software (Goble 2014, Taschuk and Wilson 2017, Arvanitou *et al.* 2021). The scientific ecosystem would benefit from funding earmarked for maintenance and dedicating time to it in project proposals. On a positive note, a few agencies, such as the Chan Zuckerberg Initiative Essential Open Source Software for Science fund (https://chanzuckerberg.com/eoss/), and the Schmidt Futures through their Virtual Institute of Scientific Software (Matthews 2022), have recognized this missed opportunity and are starting to address the lack of funding in this aspect. The scientific community and other funding agencies should welcome the efforts of maintaining original software and encourage its updates. Rather than promoting the development of replacement software that suffers from immaturity, a lack of community knowledge, and the risk of remaining unmaintained, they should focus on supporting the enhancement of established existing tools. We strongly advocate for sustainable funding to maintain existing scientific software and recognize software that adheres to best practices.

## Data Availability

No new data were generated or analysed in support of this research.
